# Cold Enrichment Methods for the Detection of Foodborne Yersiniosis: Friend or Foe?

**DOI:** 10.3390/pathogens11020278

**Published:** 2022-02-21

**Authors:** Yuwei Zhang, Stephen L. W. On

**Affiliations:** Department of Wine, Food and Molecular Biosciences, Lincoln University, P.O. Box 85054, Lincoln 7647, New Zealand; yuwei.zhang@lincolnuni.ac.nz

**Keywords:** *Yersinia*, isolation, virulence plasmid

## Abstract

*Yersinia enterocolitica* and *Y. pseudotuberculosis* are important causes of enteric illness worldwide. Rapid response to suspected foodborne outbreaks is hampered by the widespread use of cold enrichment methods that require incubation periods of 10–21 days. Although these species grow faster at elevated temperatures, part of the rationale for cold enrichment is that a key pathogenicity marker (pYV virulence plasmid) is said to be lost at elevated temperatures. Experimental data on this claim seems scarce. We previously described an approach involving an enrichment step at 37 °C for *Yersinia* detection, applied this approach to additional strains, and examined the presence of plasmids in reisolates, as well as those recovered in our original study. Plasmids were recovered from every reisolate examined; the presence of marker genes *yad*A and *vir*F denoted the virulence plasmid in 10 of the 11 strains examined. Use of an enrichment step at 37 °C does not appear to promote loss of the pYV or other plasmids harboured by foodborne pathogenic *Y. enterocolitica* and *Y. pseudotuberculosis;* wider adoption of this approach may assist the development of more rapid detection methods.

## 1. Introduction

In several countries (including New Zealand), the enteric disorder yersiniosis caused by *Yersinia enterocolitica* or *Y. pseudotuberculosis* is among the most frequently reported foodborne diseases, representing a substantive public health burden [1,2,3,4]. Detection of these species is hampered by poorly effective methods, most frequently involving cold (4–10 °C) enrichment approaches for prolonged (10–21 days) periods [5], that are clearly inadequate for rapid outbreak detection. While yersiniae grow more readily at higher temperatures [6], one of the reasons that such enrichment protocols are not adopted appears to be a long-standing belief that a key pathogenicity trait (virulence plasmid pYV) is lost at such temperatures [6,7]. Clearly, optimal characterisation of an outbreak caused by pathogenic *Yersinia * species may not be achieved where such an important virulence trait is lost, especially when pathogenicity genes located on the plasmid can be targets for PCR detection approaches [5]. 

We recently described a novel, rapid (39 h) detection approach for *Yersinia * spp. in pork mince using an initial enrichment step at 37 °C combined with non-destructive, semi-automated isolate identification using Elastic Light Scatter analysis [8]. One of the anonymous referees for this paper explicitly questioned our method, declaring that “pYV could be lost and the colony morphology might be greatly affected.” The reviewer furthermore suggested “Most colonies obtained from the unadulterated nutrient-enrichment method, may not be pathogens.” Such a viewpoint is clearly widespread: an extensive review of *Yersinia * detection methods does not describe a single culture approach utilising any temperature higher than 30 °C [5].

In this paper, we investigate the impact of our protocol on pYV plasmid carriage, with special reference to the enrichment temperature of 37 °C used.

## 2. Results and Discussion

### 2.1. Plasmid Carriage in Y. enterocolitica and Y. pseudotuberculosis Strains Recovered Using the 37 °C + 28 °C Enrichment Protocol

**Figure 1:**
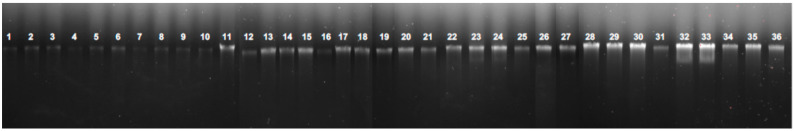
Plasmid analyses of strains examined. Labels 1–36 are strains as follows. 1, ERL 10782 (original strain). 2, Reisolate #11. 3, Reisolate #12. 4, Reisolate #17. 5, Reisolate #28. 6, Reisolate #87. 7, Reisolate #55. 8, Reisolate #56. 9, Reisolate. #68. 10, Reisolate #87B. 11, Reisolate #105. 12, ERL 110237 (original strain). 13, Reisolate #4. 14, Reisolate #19. 15, Reisolate #90. 16, Reisolate #1A. 17, Reisolate #8. 18, Reisolate #10. 19, ERL 112277. 20, ERL 032122. 21, ERL 032123. 22, ATCC 27729. 23, EWP5. 24, PT18-1. 25, ATCC51871. 26, NCTC 11174. 27, PB1+. 28, ERL 112277 reisolate. 29, ERL 032122 reisolate. 30, ERL 032123 reisolate. 31, ATCC 27729 reisolate. 32, EWP5 reisolate. 33, PT18-1 Reisolate. 34, ATCC51871 reisolate. 35, NCTC 11174 reisolate. 36, PB1+ reisolate.

Sixteen isolates representing one strain each of *Y. enterocolitica* and *Y. pseudotuberculosis* recovered from our original seeding experiments [8], and nine additional strains (see Section 3, Materials and Methods) subjected to the 37 °C + 28 °C enrichment protocol described, were examined for plasmid carriage using the QIAPrep spin miniprep assay. Plasmids were detected in every isolate examined both pre- and post-enrichment (Figure 1). 

### 2.2. PCR Analysis for Virulence Genes

The presence of *yad*A and *vir*F genes (markers of the pYV virulence plasmid) was determined by PCR (see Section 3, Materials and Methods). In 10 of the 11 strains examined, both genes were detected in plasmid preparations for each of these strains, thus indicating presence of the pYV plasmid (Supplementary Figure S1, Table 1); in four of the strains examined, these virulence genes were not detected in the simple heated lysates examined. For strain ERL 10782 (*Y. enterocolitica*), neither *yad*A nor *vir*F were detected by PCR, indicating that the plasmid detected was not pYV.

### 2.3. Discussion

The widespread use of the cold enrichment approach to the detection of foodborne *Yersinia * species may, in part, be due to statements in influential reference books that state “virulent plasmid-containing strains of *Y. pestis*, *Y. pseudotuberculosis*, and *Y. enterocolitica* rapidly become avirulent when grown at 37 °C, which results in the loss of the virulence plasmid” [6]. Several articles also refer to this feature [7,9,10]; however, specific experimental studies appear scarce. In one study that explicitly investigates the loss of the virulence plasmid in these species when cultured at 37 °C, the process is described as “spontaneous” rather than an event that can be reliably and repeatedly determined [7]. Elevated NaCl and calcium concentrations as well as acidic (pH 4–6) conditions have also been shown to stabilise plasmid carriage [10,11]. However, the conditions we used involve NaCl concentrations of 0.5%, no additional calcium and, in the original study involving a modified Ossmer broth, a pH of 8.5 [8]. In every strain of *Y. enterocolitica* and *Y. pseudotuberculosis* we examined, plasmids were detectable from colonies examined after enrichment at 37 °C and recovery on solid media at 28 °C. In 10 of 11 strains, the plasmid was presumed to be the virulence pYV type, on the basis of the presence of key genes *yad*A and *vir*F in plasmid preparations (failure of some lysates to yield a PCR product was attributed to inhibitors that may be present in such crude preparations) (Figure S1). This suggests that the risk of strains of these foodborne pathogenic species losing this key virulence trait is low under such conditions. Conversely, such conditions are conducive to more rapid growth, enabling detection in periods as low as 39 h [8]. Our observation also aligns with the view that key virulence factors including *yad*A are expressed at 37 °C, and that the genetic structure of the plasmid is designed for optimal expression at this temperature [9,12]. It seems counter-intuitive that a key virulence trait is lost at the same temperature at which it is expressed.

The observation that one of the Y. enterocolitica strains examined by us from human diarrhoea (ERL 10782) contains a plasmid that does not appear to be the pYV variant, since neither *yad*A or *vir*F were detected. Although pYV is regarded as a key virulence trait, it is not the only one, and virulent strains are known that lack this plasmid [13].

The use of low temperatures to preserve the *Yersinia * virulence plasmid is not the only rationale underpinning the widespread use of cold enrichment to recover these organisms from foods. Foodborne pathogenic *Yersinia * species may be found in foods that harbour many other bacterial species in higher numbers that may outcompete the growth of yersiniae, where higher incubation temperatures are used [9,12]. However, the apparent dominance of cold enrichment approaches to detect *Yersinia * in foods may be deterring researchers from exploring other approaches to enrich and/or detect these bacteria from complex matrices in a more rapid manner. We hope our study serves to demonstrate that loss of the pYV virulence factor appears to be of minimal risk when recovering *Yersinia * species at temperatures more conducive to organismal growth. The value of such alternatives has already been demonstrated [8]; we hope other researchers will further examine the opportunity to develop better tools for an important cause of foodborne illness worldwide.

## 3. Materials and Methods

### 3.1. Strains Used

Original cultures, as well as reisolates of strains ERL 10782 (*Y. enterocolitica*) and ERL 110237 (*Y. pseudotuberculosis*) used to seed pork mince samples in the original study [8], were studied. Additional strains of *Y. enterocolitica* (ERL 112277, ERL 032122, ERL 032123, EWP5, PT18-1, ATCC27729, ATCC51871, NCTC11174) and *Y. pseudotuberculosis* (PB1+) were also examined.

### 3.2. Culture Methods

For original cultures and reisolates of the seeding strains, isolates were subcultured twice from frozen stocks of these cultures on 5% blood agar (Fort Richard, Auckland, New Zealand) and incubated overnight at 28 °C. Thereafter, a single colony was picked and inoculated into 25ml nutrient broth (Oxoid no. 2, Basingstoke, UK) and cultured overnight at 37 °C with shaking at 100 rpm. Thereafter, 50µL of the enrichment was taken and inoculated onto Tryptone Soya Agar (Fort Richard, Auckland, New Zealand) for 22 h at 28 °C as before [8]. Single colonies from each culture were then taken and subjected to plasmid and PCR analyses as described below.

### 3.3. Plasmid DNA Isolation and Detection

The extraction of plasmid DNA from the bacterial samples was achieved using a commercial kit (QIAprep® Spin Miniprep Kit, QIAGEN, Hilden, Germany) in accordance with the manufacturer’s instructions. For detection, 10 μL pDNA was loaded onto a 0.8% agarose gel and electrophoresed in 0.5× TBE buffer at 90 V for 90 min. Plasmid visualisation was achieved by staining with SYBRTM Safe DNA Gel Stain (Invitrogen, Thermo Fisher Scientific, Waltham, MA, USA).

### 3.4. PCR Analysis

The presence of key virulence marker genes *yad*A and *vir*F was determined by PCR using methods described previously [14], with minor modifications. In brief, the primers used were: for *yad*A (amplicon size 849 bp), *yad*A-F (CTTCAGATACTGGTGTCGCTGT) and *yad*A-R (ATGCCTGACTAGAGCGATATCC); and for *vir*F (amplicon size 561bp), *vir*F-F (GGCAGAACAGCAGTCAGACATA) and *vir*F-R (GGTGAGCATAGAGAATACGTCG). PCR reactions were undertaken on both plasmid extractions (described above) and heated lysates of colonies to examine the possibility that virulence genes may be chromosomally borne, were they not detected in plasmid preparations. Assays were performed in a 20 μL reaction system comprising repliQa HiFi ToughMix® (Quantabio, Beverly, MA, USA): 10 μL, Forward primer: 1 μL, Reverse primer: 1 μL, Nuclease-free water: 6 μL and Template: 2 μL. The concentration of pDNA stocks and cell lysates were determined by a DeNovix® DS-11+ Spectrophotometer (DeNovix Inc., Wilmington, DE, USA) and adjusted to 50 ng/μL and 100 ng/μL, respectively, with UltraPure™ DNase/RNase-free distilled water (Thermo Fisher Scientific Ltd.). Hence, the starting DNA amount in each of the 20 μL pDNA or heated cell lysate PCR assays was 100 ng and 200 ng, respectively, to make the final DNA concentration in the reaction mixture at 5 ng/μL for those that contained pDNA, and 10 ng/μL for those that contained simple heated cell lysates. Products were detected by electrophoresis in a Wide mini-sub cell system (Bio-Rad, Hercules, CA, USA) using 5 μL of each sample loaded onto a 1.5% agarose gel and electrophoresed for 90 min at 90 V in 0.5× TBE buffer.

## Figures and Tables

**Table 1:** Summary of plasmid, *yad*A and *vir*F gene detections by PCR among *Yersinia* strains examined. PCR results are presented for whole-cell heated lysates and plasmid preparations separately.

Strain No.	Plasmid	*yad*A Plasmid	*yad*A Lysate	*vir*F Plasmid	*vir*F Lysate	Species
ERL 10782 *	+	-	-	-	-	Ye
Reisolate #11	+	-	-	-	-	Ye
Reisolate #12	+	-	-	-	-	Ye
Reisolate #17	+	-	-	-	-	Ye
Reisolate #28	+	-	-	-	-	Ye
Reisolate #87	+	-	-	-	-	Ye
Reisolate #55	+	-	-	-	-	Ye
Reisolate #56	+	-	-	-	-	Ye
Reisolate #68	+	-	-	-	-	Ye
Reisolate #87B	+	-	-	-	-	Ye
Reisolate #105	+	-	-	-	-	Ye
ERL 110237 *	+	+	+	+	+	Yp
Reisolate #4	+	+	+	+	+	Yp
Reisolate #19	+	+	+	+	+	Yp
Reisolate #90	+	+	+	+	+	Yp
Reisolate #1A	+	+	+	+	+	Yp
Reisolate #8	+	+	+	+	+	Yp
Reisolate #10	+	+	+	+	+	Yp
ERL 112277	+	+	-	+	-	Ye
ERL 032122	+	+	+	+	+	Ye
ERL 032123	+	+	-	+	+	Ye
ATCC 27729	+	+	+	+	+	Ye
EWP5	+	+	-	+	-	Ye
PT18-1	+	+	-	+	-	Ye
ATCC51871	+	+	+	+	+	Ye
NCTC 11174	+	+	+	+	+	Ye
PB1+	+	+	+	+	+	Yp

The original seeding cultures in reference [8] are denoted by an asterisk, *. Reisolates refer to strains of that strain recovered after pork mince seeding studies [8] or experiments described in this paper. +, trait detected; -, trait not detected; Ye, *Y. enterocolitica*; Yp, *Y. pseudotuberculosis*; ERL, Enteric Reference Laboratory of the Institute of Environmental Science, New Zealand; ATCC, American Type Culture Collection, Virginia, MA, USA; NCTC, National Type Culture Collection, London, UK. The strain prefixes of EWP, PT and PB are of unknown affiliation and are reproduced as received.

## Data Availability

All relevant data are published here or in the supplementary figure to this article, available online.

## Supplementary Materials

The following supporting information can be downloaded at: https://www.mdpi.com/article/10.3390/pathogens11020278/s1, Figure S1: PCR analyses of heated lysates and purified plasmid DNA for the presence of virulence marker genes *yad*A and *vir*F.
