# Supplementary material for: Cold Enrichment Methods for the Detection of Foodborne Yersiniosis: Friend or Foe?
Source: Pathogens. 2022 Feb 21;11(2):278. doi: 10.3390/pathogens11020278 (PMC8875810; doi:10.3390/pathogens11020278)
Supplement: Supplementary file 1 [file pathogens-11-00278-s001.zip › pathogens-1602673-supplementary.pdf]

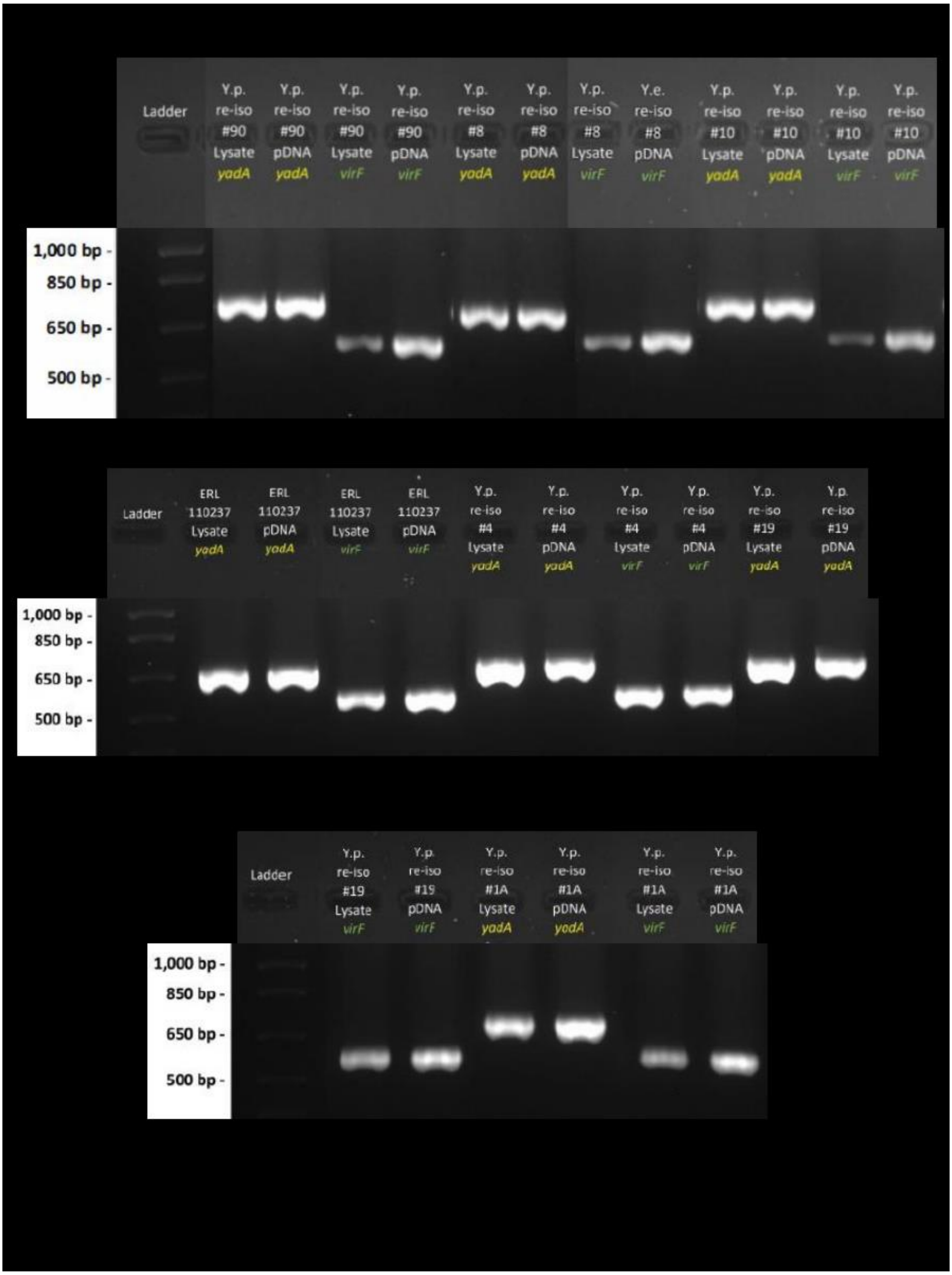
Ladder
Y.p. re-iso #90 Lysate yadA
Y.p. re-iso #90 pDNA yadA
Y.p. re-iso #90 Lysate virF
Y.p. re-iso #90 pDNA virF
Y.p. re-iso #8 Lysate yadA
Y.p. re-iso #8 pDNA yadA
Y.p. re-iso #8 Lysate virF
Y.e. re-iso #8 pDNA virF
Y.p. re-iso #10 Lysate yadA
Y.p. re-iso #10 pDNA yadA
Y.p. re-iso #10 Lysate virF
Y.p. re-iso #10 pDNA virF
1,000 bp -
850 bp -
650 bp -
500 bp -
Ladder
ERL 110237 Lysate yadA
ERL 110237 pDNA yadA
ERL 110237 Lysate virF
ERL 110237 pDNA virF
Y.p. re-iso #4 Lysate yadA
Y.p. re-iso #4 pDNA yadA
Y.p. re-iso #4 Lysate virF
Y.p. re-iso #4 pDNA virF
Y.p. re-iso #19 Lysate yadA
Y.p. re-iso #19 pDNA yadA
1,000 bp -
850 bp -
650 bp -
500 bp -
Ladder
Y.p. re-iso #19 Lysate virF
Y.p. re-iso #19 pDNA virF
Y.p. re-iso #1A Lysate yadA
Y.p. re-iso #1A pDNA yadA
Y.p. re-iso #1A Lysate virF
Y.p. re-iso #1A pDNA virF
1,000 bp -
850 bp -
650 bp -
500 bp -

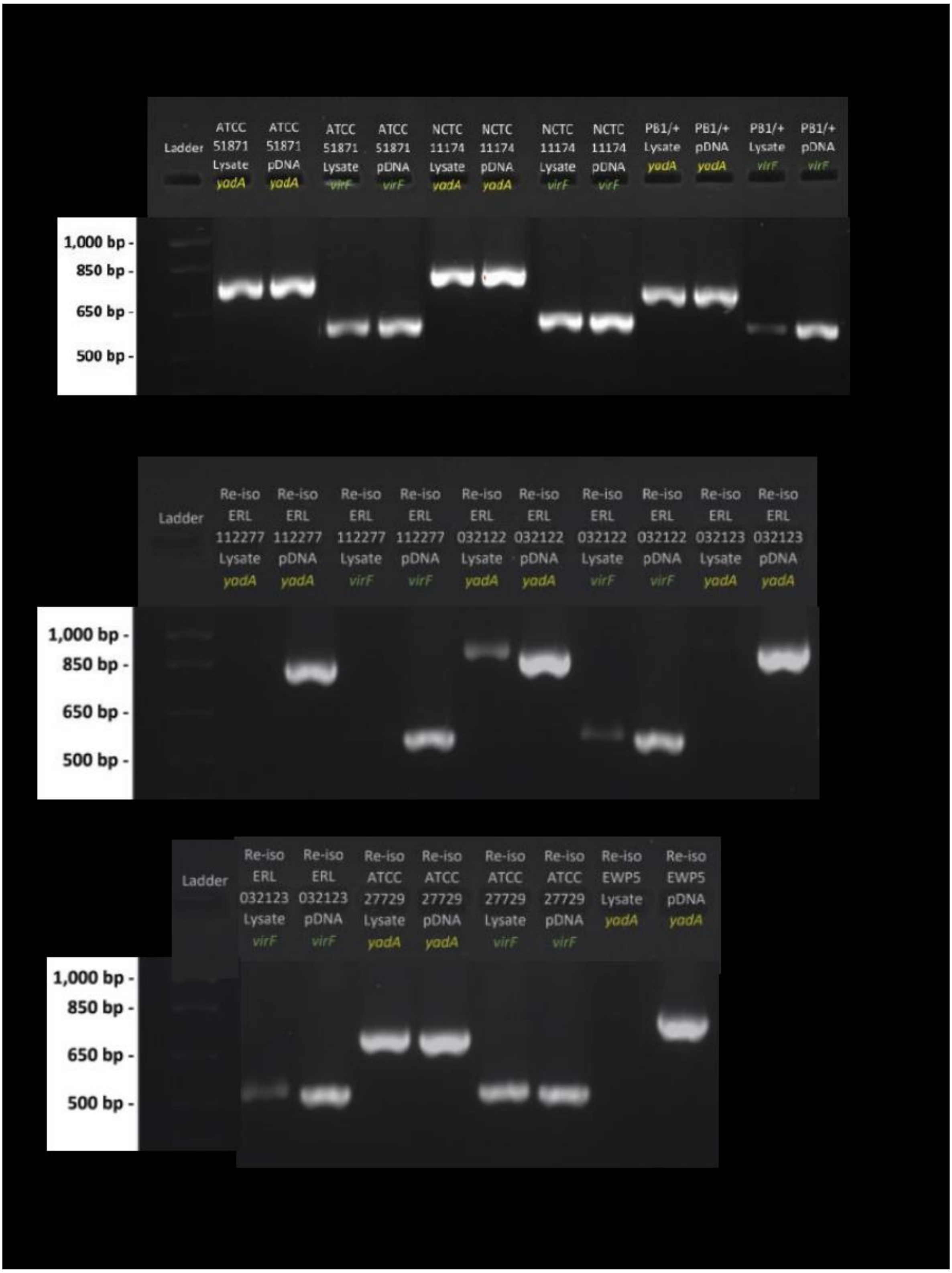
Ladder
ATCC 51871 Lysate yadA
ATCC 51871 pDNA yadA
ATCC 51871 Lysate virF
ATCC 51871 pDNA virF
NCTC 11174 Lysate yadA
NCTC 11174 pDNA yadA
NCTC 11174 Lysate virF
NCTC 11174 pDNA virF
PB1/+ Lysate yadA
PB1/+ pDNA yadA
PB1/+ Lysate virF
PB1/+ pDNA virF
1,000 bp -
850 bp -
650 bp -
500 bp -
Ladder
Re-iso ERL 112277 Lysate yadA
Re-iso ERL 112277 pDNA yadA
Re-iso ERL 112277 Lysate virF
Re-iso ERL 112277 pDNA virF
Re-iso ERL 032122 Lysate yadA
Re-iso ERL 032122 pDNA yadA
Re-iso ERL 032122 Lysate virF
Re-iso ERL 032122 pDNA virF
Re-iso ERL 032123 Lysate yadA
Re-iso ERL 032123 pDNA yadA
1,000 bp -
850 bp -
650 bp -
500 bp -
Ladder
Re-iso ERL 032123 Lysate virF
Re-iso ERL 032123 pDNA virF
Re-iso ATCC 27729 Lysate yadA
Re-iso ATCC 27729 pDNA yadA
Re-iso ATCC 27729 Lysate virF
Re-iso ATCC 27729 pDNA virF
Re-iso EWP5 Lysate yadA
Re-iso EWP5 pDNA yadA
1,000 bp -
850 bp -
650 bp -
500 bp -

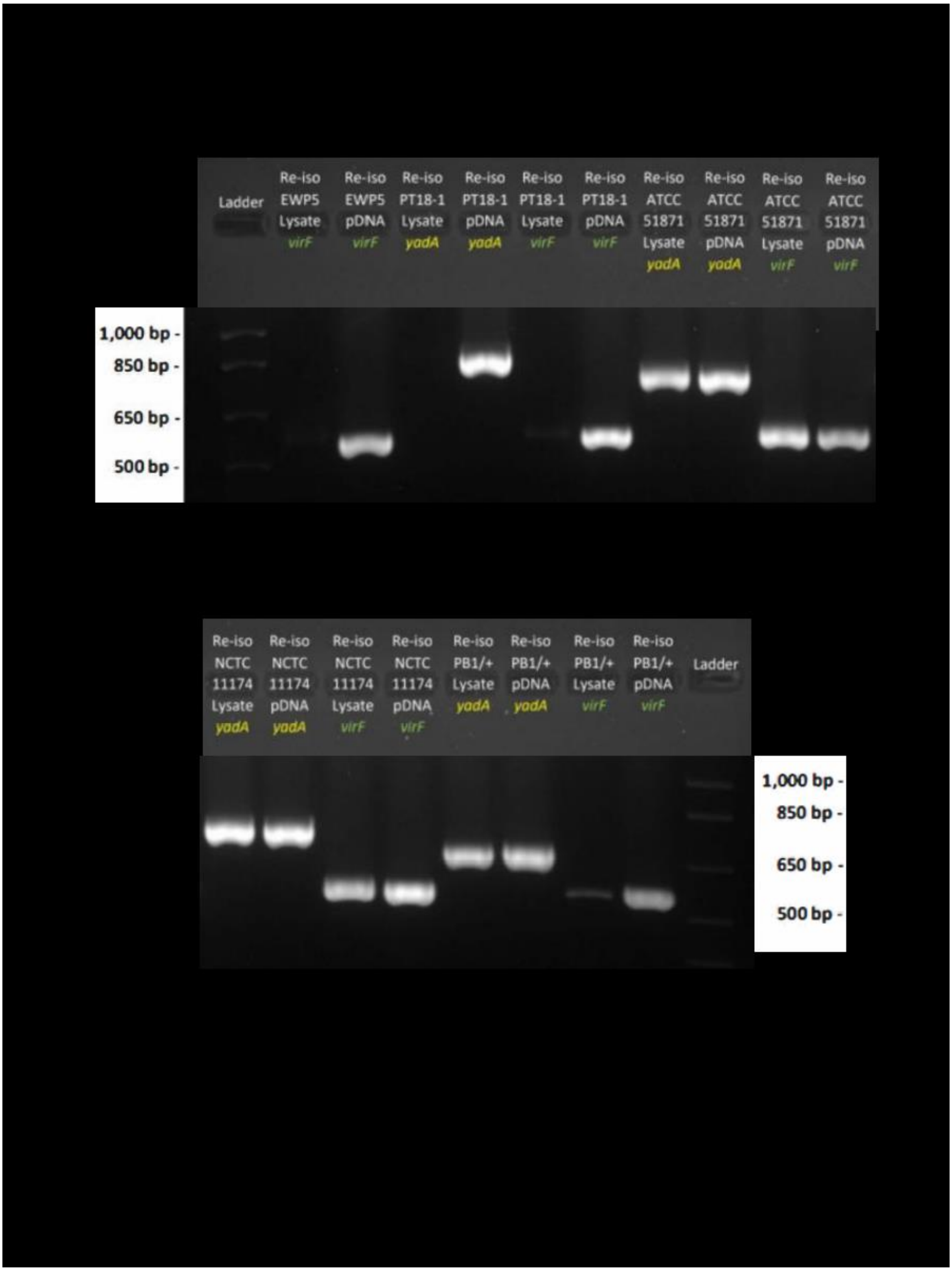

Figure S1. PCR analyses of heated lysates and purified plasmid DNA for the presence of virulence marker genes *yadA* and *virF*.
